# Ethynilestradiol 20 mcg plus Levonorgestrel 100 mcg: Clinical Pharmacology

**DOI:** 10.1155/2014/102184

**Published:** 2014-11-16

**Authors:** Stefano Lello, Andrea Cavani

**Affiliations:** ^1^Gynecological Endocrinology, Pathophysiology of Menopause and Osteoporosis, IDI-IRCCS, Via Monti di Creta 104, 00167 Rome, Italy; ^2^Laboratory of Experimental Immunology, IDI-IRCCS, 00167 Rome, Italy

## Abstract

Estroprogestins (EPs) are combinations of estrogen and progestin with several actions on women's health. The different pharmacological composition of EPs is responsible for different clinical effects. One of the most used low-dose EP associations is ethinylestradiol 20 mcg plus levonorgestrel 100 mcg in monophasic regimen (EE20/LNG100). This review summarizes clinical pharmacology, cycle control, and effects on lipid and glucose metabolism, coagulation, body weight/body composition, acne, and sexuality of EE20/LNG100. Overall, EE20/LNG100 combination is safe and well tolerated, and in several studies the incidence of adverse events in the treated group was comparable to that of the placebo group. Cycle control was effective and body weight/body composition did not vary among treated and untreated groups in most studies. The EE20/LNG100 combination shows mild or no effect on lipid and glucose metabolism. Lastly, EE20/LNG100 is associated with a low risk of venous thromboembolism (VTE). In conclusion, in the process of decision making for the individualization of EPs choice, EE20/LNG100 should be considered for its favorable clinical profile.

## 1. Introduction

Estroprogestins (EPs) are pharmaceutical compounds containing estrogen and progestin. Existing progestogen compounds can be classified as first- (e.g., norethisterone, norethindrone, ethynodiol diacetate, and lynestrenol), second- (levonorgestrel and norgestrel), and third-generation (desogestrel, gestodene, and norgestimate).

Estrogen can decrease follicle-stimulating hormone and luteinizing hormone, even if firstly it was added to progestin to reduce or avoid symptoms which follow ovarian blockage and to improve cycle control. The roles of progestin are to decrease luteinizing hormone levels through a negative feedback mechanism, to thicken cervical mucus, and to decrease the endometrial proliferation after estrogen mitotic stimulation. Notably, these activities of EPs are used not only for contraception, but also as a tool to obtain a better health profile in many women, in the so-called “noncontraceptive use.” In this view, EPs can be considered as an important tool for women's health, due to the effects of EPs on menstrual pain, excessive menstrual bleeding, endometriosis, polycystic ovary syndrome (PCOS), and the protection against some cancers (ovary, endometrium, and colon) [1].

Different EPs can show different clinical effects and different risk profile according to their specific pharmacological composition (i.e., type and dose of estrogen and progestin). One of the most used associations is ethinylestradiol (EE) 20 mcg + levonorgestrel (LNG) 100 mcg in monophasic regimen (EE20/LNG100).

This review summarizes clinical pharmacology, cycle control, and effects on lipid and glucose metabolism, coagulation, body weight/body composition, and acne of the EE20/LNG100 used once daily for 21 days of a 28-day cycle (21/7 regimen).

## 2. Selection of Evidence

Key papers for inclusion in this paper were collected by browsing MEDLINE using pertinent keywords (e.g., ethinylestradiol and levonorgestrel); papers included in the reference list of the identified manuscript could also be considered for inclusion, as well as relevant abstracts or papers from the personal collection of literature of the authors.

Papers were selected for inclusion according to their relevance for the topic, according to authors' opinion.

## 3. EE20/LNG100: Clinical Pharmacology

### 3.1. Ethinylestradiol

**Figure 1:**
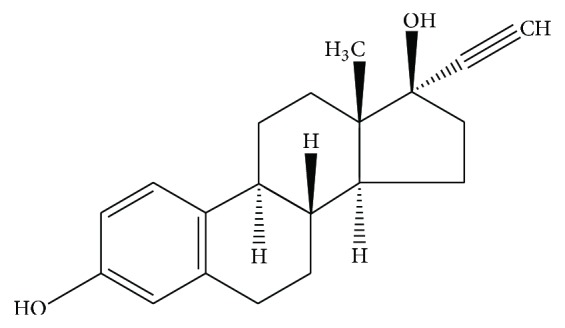
Structural formula of the 17*β*-estradiol derivative ethinylestradiol (EE).

EE is the most used estrogen in EPs. It is more potent than estradiol, due to the presence of the 17*α*-ethinyl group, which can prevent the oxidation of 17*β*-hydroxy group (Figure 1). The 17*α*-ethinyl group can be oxidized, with the formation of an intermediate element which inhibits the cytochrome P450 isoenzymes (e.g., CYP3A4) involved in estrogen metabolism. Thus, EE can reduce its catabolism by inhibiting the hydroxylation at C2 through the blockade of these specific CYP isoenzymes [2, 3].

After oral administration, besides oxidative metabolism, EE undergoes glucuronidation and sulphatation by specific enzymes (e.g., glucuronyltransferase and sulphotransferase). The reduction of enzymatic inactivation results in the dose-dependent hepatic modulation of a series of activity, such as protein synthesis. For example, EE stimulates sex hormone binding globulin (SHBG), thyroxin binding globulin (TBG), and cortisol binding globulin (CBG) but also has effects on the production of haemostatic elements, lipids, and lipoproteins [4, 5]. The oral bioavailability of oral EE ranges from 38 to 48%, due to a high first-pass metabolism, which, in turn, determines an important interindividual variation in EE plasma levels [6]. Hepatic metabolism yields EE conjugated and metabolites circulating into blood vessels. Of the oral dose, about 1% circulates as EE and is bound by 98.5% to albumin, with EE not presenting affinity for SHBG. Enterohepatic recirculation is important in the EE pharmacokinetics (PK), and the metabolic passages are based on hydroxylation at C2 and C4, with formation of catechol estrogens, which can be metabolized into 2- and 4-methoxy-EE. EE metabolites are excreted by feces and urine.

### 3.2. Levonorgestrel

**Figure 2:**
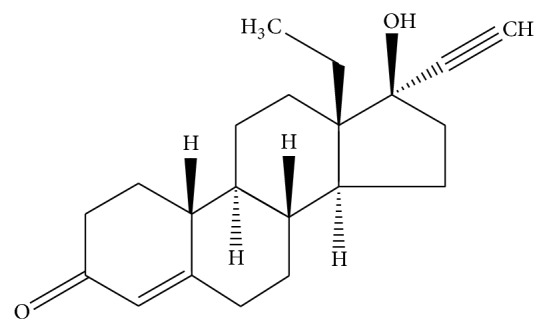
Structural formula of the progestin levonorgestrel.

LNG (Figure 2) is rapidly absorbed when administered orally. The bioavailability is about 100%, with no relevant first-pass effect, and the peak plasma-level is obtained between 1 and 3 hours after oral administration. LNG is bound to SHGB by 47.5% (this portion can be viewed as a sort of “reservoir” to maintain blood levels of LNG) and to serum albumin by 50% (more promptly available) and 2.5% is not bound. The half-life is about 15 hours [7], and level of LNG is still detectable 48 hours after the administration [8–10].

LNG has a marked progestin activity, no mineralocorticoid or glucocorticoid effects, and an antiestrogenic action at hepatic level. LNG has also a very high affinity for the uterine progesterone receptor [11]. The reduction of the Δ4-3-keto group and hydroxylation are important metabolic pathways for LNG [12]. LNG and its metabolites (glucurono- and sulphoconjugated) are excreted by urine and feces. The lowest ovulation inhibiting dose of LNG is 50 mcg/day [13]. Data from animal models show that the dose stimulating a weight increase in the ventral prostate is >100-fold greater than the dose needed to inhibit ovulation; moreover, very little progestin is required to inhibit ovulation, even when used alone rather than in combination with an estrogen [14]. These results suggest the important antigonadotropic effect exerted by LNG. Moreover, LNG has a very high relative binding affinity for progesterone receptor [15], thus suggesting a strong progestin action.

One study [16] investigated the PK of EE20/LNG100 in 18 young, healthy women. Serum levels of EE and LNG were assayed after single and repeated daily oral doses during three cycles (21/7 regimen). Serum maximum concentration was reached, for both EE and LNG, between 1 and 2 hours after single and repeated administration on a daily basis. The serum concentration of EE increased after multiple daily administrations, with about twofold accumulation. In addition, serum concentrations of LNG increased following repeated administrations, with steady-state being reached after 11 days since the intake of the first tablet. By comparing the AUC 0–24 values after the first and the last tablet, LNG showed an accumulation by a factor of 3 during a cycle of treatment. The PKs for steady state of LNG were similar after the end of the first and the third cycle of administration, thus indicating no further accumulation over a long-term intake. The clearance and distribution volume of LNG decreased and half-life increased after multiple daily administration.

In conclusion, from a pharmacological point of view, LNG is a potent progestin with an important antiestrogenic action [17] also at hepatic level (as shown by its effects on SHBG production, e.g., the ability of LNG to partially counteract the EE-induced SHBG production) and with high oral bioavailability due to no relevant first-pass effect, thus providing lower interindividual bioavailability variations.

## 4. EE20/LNG100 and Ovulation Inhibition

Adequate suppression of ovarian activity with EE20/LNG100 was first shown in an ovulation inhibition study on three treatment cycles [18] with a highly-sensitive study design [19]. Mean levels of LH, FSH, 17beta-estradiol, and progesterone were suppressed during treatment, with a normal ovulation restored in posttreatment cycles; these results were confirmed also by an ultrasound examination. In another study, the rapid restoration of ovarian activity was confirmed by mean serum progesterone levels [20].

## 5. EE20/LNG100 Effects on Lipids

Total and HDL-cholesterol, high-density lipoprotein subfraction-2 (HDL-2), and apolipoprotein A-I did not significantly change from baseline during a 24-month study performed on 28 women (age range: 19–44 years) [21]. In addition the HDL-2/HDL-3 ratio did not change significantly. In the same study, between cycles 3 and 18, there were statistically significant increases versus baseline in LDL-cholesterol (*P* ≤ 0.05), triglycerides (*P* ≤ 0.01), apolipoprotein-B (*P* ≤ 0.001), the ratio LDL/HDL (*P* ≤ 0.05), total cholesterol/HDL (*P* ≤ 0.05), and the ratio apolipoprotein-B/apolipoprotein A-I (*P* ≤ 0.05). Interestingly, even if single subject values were reported occasionally outside the normal reference range, there were no subjects with relevant alterations in the lipid pattern, and the changes in lipid profile were similar to those observed with other low-dose EPs. Lipid changes were no longer significant at 24 months.

Reisman et al. [22] compared EE20/LNG100 with a triphasic EP combination containing EE 35 mcg plus 500, 750, and 1000 mcg norethindrone (NET). While changes from baseline in triglycerides levels were not different between the two EPs, the mean increase in cholesterol level was significantly lower in the EE20/LNG100 group (0.203 mmol/L) than in the EE35/NET 500-750-1000 group (0.475 mmol/L; *P* < 0.05).

An open-label, randomized study [23] compared EE20/LNG100 with EE30/LNG150 over a 1-year period of observation in 48 subjects, showing a decrease for HDL-2 cholesterol and lipoprotein(a) and an increase for LDL cholesterol, VLDL cholesterol, and total triglycerides in both groups from baseline to the 13th treatment cycle. Interestingly, the wide majority of lipid values remained within the normal reference range. Moreover, there was a trend to have lower changes in the EE20/LNG100 group than in EE30/LNG150 group.

Another study, by Endrikat et al. [24], compared the combination EE 20 mcg + LNG 100 mcg with EE 30 mcg + LNG 150 mcg in terms of effects on lipids, carbohydrates, and coagulation during a 13-cycle period. The lower dosage of EE/LNG (EE20/LNG100) showed a milder impact on lipids and carbohydrates in comparison with the EE30/LNG150. Overall, lipid changes were more favorable for the combination EE20/LNG100 versus EE30/LNG150.

Thus, the impact on lipid levels shown with EE20/LNG100 is globally mild with values usually within the normal range values.

## 6. EE20/LNG100 and Carbohydrate Metabolism

Endrikat et al. [24] compared the effects on carbohydrate metabolism of EE20/LNG100 and EE30/LNG150. Overall, carbohydrate metabolism was not significantly changed, and the variations were lower in EE20/LNG100 group than EE30/LNG150 group; in particular, fasting levels of glucose, insulin, and C-peptide were decreased with EE20/LNG100, and the 3-hour area under the curve (AUC 0–3 h) showed a decrease in EE20/LNG100 (−1635.0 pmol/L × min) group. On the other hand, there was an increase in EE30 mcg/LNG150 mcg group, with a significant difference between the two groups (*P* < 0.04).

Also the study by Skouby et al. [23] compared EE20/LNG100 and EE30/LNG150; the median values for the fasting levels of insulin and C-peptide slightly increased or remained unchanged, while the fasting glucose levels slightly decreased after 13 treatment cycles (% variation from baseline to cycle 13: −15.8 for EE20/LNG100 and −18.0 for EE30/LNG150). With regard to the AUC 0–3 h, for glucose, the variation was similar in both groups during the oral glucose tolerance test (OGTT) (median absolute change from baseline to cycle 13: −59 mmol/(L min) for both groups), while the insulin AUC 0–3 h was less increased in the EE20/LNG100 group (+4940 pmol/(L min)) than in the EE30/LNG150 group (7373 pmol/(L min)). No significant differences between the treatment groups for any of the carbohydrate metabolism variables were disclosed.

## 7. EE20/LNG100, Body Weight, and Body Composition

Weight gain is one of the most common reasons for discontinuation of EPs [27–29]. Even if not confirmed by several studies, the perception of this problem remains in clinical practice among patients and, sometimes, among clinicians. On the other hand, a patient's perception of weight gain (an actual weight gain or “a sensation of weight gain”) can lead to a decreased compliance, with a subsequent increased rate of misuse and discontinuation of EPs use.

A study by Hite et al. [30] reported no weight change or weight reduction in 75% of the subjects with EE20/LNG100 in a 6-cycle study.

Another interesting 6-cycle study [31], in which the combination EE20/LNG100 was compared with placebo, showed no difference in body weight changes between EP and placebo; in particular, there were no differences among the proportion of patients with weight gain (≥1 kg), no weight change (<1 kg), or weight loss (≥1 kg).

**Figure 3:**
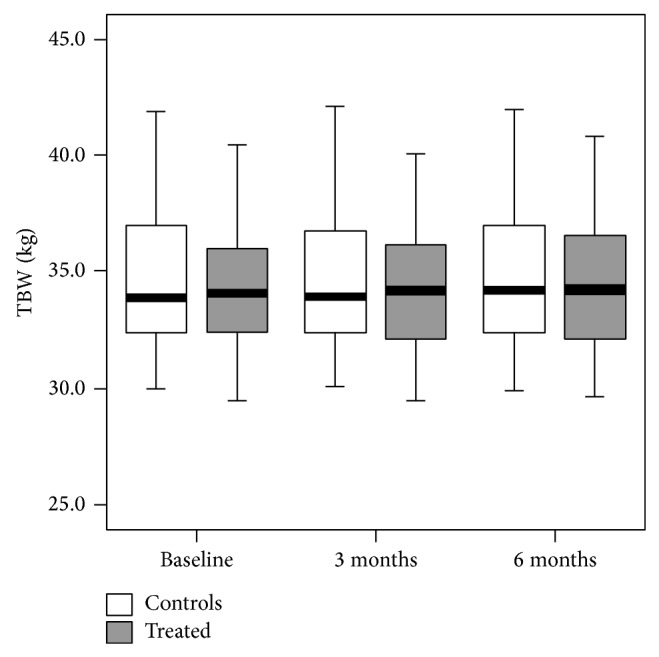
Total body water (TBW) showed no significant differences between EP-treated and control subjects or within each group [25].

Lello et al. [25] evaluated the effects of EE20/LNG100 on body weight and body composition (this latter assessed by bioelectrical impedance) in a 6-month study on 47 subjects treated with this EP and 31 controls (no hormone intake). EE20/LNG100 combination did not significantly change body weight, body mass index, and waist/hip ratio in comparison with nontreated subjects. More interestingly, in terms of body composition, the combination had no impact on fat mass, fat-free mass, total body water, intracellular water, and extracellular water versus baseline and versus lack of treatment (Figure 3).

Endrikat et al. [24] reported that 87.9% of the women treated with EE20/LNG100 maintained a constant body weight (±3 kg), while 9.4% of patients had a loss >3 kg of body weight.

In a randomized, multicenter, and placebo-controlled trial using EE20/LNG100 as an active treatment for six cycles in moderate acne treatment [32], changes in body weight were similar between EE20/LNG100 group and placebo group.

## 8. EE20/LNG100: Cycle Control, Safety, and Tolerability

In a 6-cycle study [33] on 792 women (age range: 17–49 years) the effect of EE20/LNG100 on cycle control was evaluated. There was an incidence (% of the cycles; total number of cycles valid for analysis: 7508) of 4.3% for breakthrough bleeding (BTB), 12.1% for spotting, 11% for BTB + S, and 2.6% for amenorrhea. The mean length of withdrawal bleeding was 4.8 days (range between 3 and 7 days in 86% of cycles). The mean bleeding intensity was generally reported as mild. In the same study, >97% of the women showed normal blood pressure (systolic ≤ 140 mmHg; diastolic ≤ 90 mmHg) at baseline and during the observation. With regard to the most common side effects considered possibly drug related, headache was reported in 14% of the subjects, metrorrhagia in 8%, dysmenorrhea in 7%, and nausea in 7%; moreover, abdominal pain was pointed out in 4%, breast pain in 4%, emotional lability in 3%, acne in 3%, depression in 2%, amenorrhea in 2%, and vaginal moniliasis in 2%. A total number of 131 (8%) women reported an adverse event as a reason for discontinuation, the most frequent drug-related event being headache and metrorrhagia (1%); less frequent reasons for discontinuation (<1%) were amenorrhea, depression, emotional lability, hypertension, acne, menorrhagia, nausea, hypercholesterolemia, weight gain, dysmenorrhea, and flatulence. No relevant cardiovascular events were reported during the study. Thus, in this study EE20/LNG100 was well tolerated and showed an overall good cycle control.

**Figure 4:**
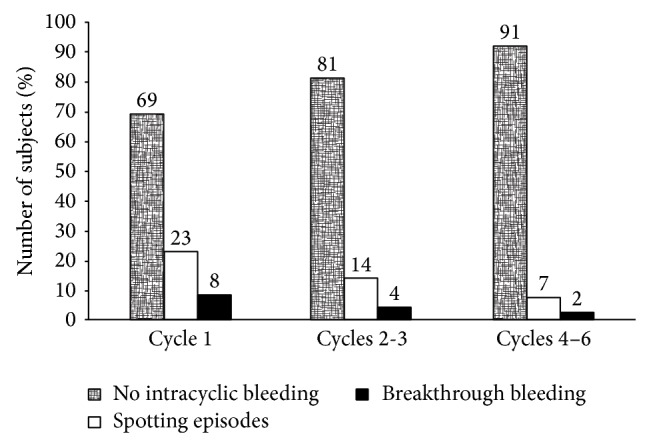
Analysis of cycle control parameters: percentage of subjects with no IMB, S episodes, or BTB. Modified from [26].

Endrikat et al. [26] compared cycle control and tolerability between two EP combinations containing EE 20 mcg associated with 100 mcg LNG or 500 mcg norethisterone (NET). The results from these two preparations were compared with a standard preparation containing EE 30 mcg + LNG 150 mcg. In this study, while cycle control was good with the two combinations with LNG, less favorable profile was obtained with the EP containing NET. In particular, the proportion of women with spotting or BTB was significantly lower with EE20/LNG100 and EE30/LNG150 than with EE20/NET500 (Figure 4). Comprehensively, spotting was present in 9.3% of the cycles with EE20/LNG100, in 21% of the cycles with EE20/NET500, and in 3.3% in the cycles with EE30/LNG150; BTB overall incidence in all 13 cycles of observation was 4.1% for EE20/LNG100, 11.7% for EE20/NET500, and 1.0% for EE30/LNG150. As for intermenstrual bleeding (IMB), in 87% of all cycles with EE20/LNG100 IMB was not reported, while the percentage was 67.6% and 95.5% with EE20/NET500 and EE30/LNG150, respectively. Moreover, the incidence of IMB decreased from 18.4% (baseline) in the EE20/LNG100 group to 7.7% in cycle 13 in the study. Amenorrhea was reported more frequently in the first cycles of observation and then decreased over time. The incidence over the study (13 cycles) was 7.1% with EE20/LNG100, 20.6% with EE20/NET500, and 0.9% with EE30/LNG150. Dysmenorrhea improved during this study, the incidence being highest at baseline and decreasing to 2.7% for EE20/LNG100, 5.1% for EE20/NET500, and 5.5% for EE30/LNG150, without significant differences among groups. Over 13 cycles of observation, a low incidence of drug-related side effects was reported for EE20/LNG100 group, with headache, breast tension, and nausea being the most frequent; nevertheless, only 7% of women in the EE20/LNG100 group discontinued the treatment due to an adverse event at the end of the study. Blood pressure was not modified significantly during the treatment with EE20/LNG100; 5.3% of the women taking EE20/100LNG showed individual systolic blood pressure occasionally >140 mmHg, and 3.4% subjects in the same group had diastolic blood pressure >90 mmHg. Thus, also in this study the combination EE20/LNG100 showed good cycle control and tolerability. Good cycle control for EE20/LNG100 was reported also in other studies [22, 30, 34].

In the study by Coney et al. [31], a similar percentage of women in the EE/LNG (82.0%) and placebo (76.9%) groups reported one or more adverse events (*P* = 0.11). The percentage of women in the EE/LNG and placebo groups who experienced possibly estrogen-related side effects, such as headache, migraine, nausea, vomiting, breast pain, and weight gain, was not different.

In a study on 1708 subjects (age range: 17–49 years) observed for 26,554 cycles, the most common adverse events reported as reasons for EE20/LNG100 discontinuation were headache (2% of subjects) and metrorrhagia (2%) [35].

Another study [34] on 805 women (age range: 18–36 years) treated for 4400 treatment cycles reported no serious adverse events that are treatment related, with headache reported by 17.3% of the women, breast tension by 11%, and nausea by 7.7%. No clinically relevant changes in laboratory findings, blood pressure, and body weight were reported.

## 9. EE20/LNG100, Hemostasis, and Venous Thromboembolism (VTE)

Archer et al. studied the effects on hemostasis of EE20/LNG100 in 30 healthy women (mean age: 29.9 ± 5.1) over a 12-cycle period of observation [36]. Factor X increased significantly from baseline during cycles 3 and 6 (*P* < 0.001) and cycle 12 (*P* < 0.01), whereas a significant (*P* < 0.05) decrease in factor VII concentration was seen at cycle 3. In particular, the coagulation activation marker thrombin-antithrombin (TAT) complex did not change significantly during the study. At cycles 3, 6, and 12 total protein S and antithrombin antigen levels (*P* < 0.001) decreased from baseline. Protein S activity decreased (*P* < 0.05) from baseline at cycles 3 and cycle 6 but was no longer different from baseline at cycle 12. Antithrombin activity or free protein S antigen did not show any significant changes from baseline during the study. Plasminogen antigen and activity levels increased significantly (*P* < 0.001) during the observation, while fibrinogen was not significantly modified. D-dimer increased significantly at cycles 3, 6, and 12, with a smaller increase at cycle 12. Altogether, only sporadic individual values (plasminogen antigen and activity) were outside the normal reference range, and no subject showed clinically important variation in hemostatic profile. The increase of plasminogen antigen and activity were seen in this study as a manifestation of increased fibrinolysis, and the general changes in haemostatic parameters were regarded as consistent with those of other low-dose oral EPs by the authors.

Endrikat et al. [24] compared EE20/LNG100 with EE30/LNG150 in terms of effect on hemostatic variables. In the EE20/LNG100 group, the median concentration of prothrombin fragment 1 + 2 increased slightly during the study and reached, starting from a baseline value of 0.53 *μ*g/L, a value of 0.8 *μ*g/L, below the upper normal limit of the reference range (2.88 *μ*g/L). Also D-dimer did not show any significant change between baseline and cycle 13. Plasminogen (a profibrinolytic marker) was increased by 31.1% after 13 cycles, while tPA antigen (a profibrinolytic marker) was reduced by 31.1 ng/mL. On the other hand, some procoagulatory markers were increased (fibrinogen +16.1%; F VII Ag +15.5%; F VIIa +68.8%), while some anticoagulatory factors were decreased (F VII Act −10%; F VIII −6.7%), thus indicating a new balance between coagulation and fibrinolysis. Overall, hemostatic parameters showed minor changes, all within the normal range of variation, thus indicating only a new and different balance between coagulation and fibrinolysis. The median concentration of prothrombin fragments 1 + 2 (considered as a marker for changes in coagulation) did not change significantly versus baseline, while D-dimer (a marker of fibrinolysis) did so. There were no differences between EE20/LNG100 and EE30/LNG150 groups. Moreover, the changes of all hemostatic variables were all within the normal ranges.

Overall, EE20/LNG100 seems to stimulate both coagulation and fibrinolysis, with no effects on hemostatic balance.

From an epidemiological point of view, the problem of venous thromboembolism (VTE) becomes evident early after the introduction of EPs in clinical use [37]. It was suggested by some reports since the 1990s that EPs containing second-generation progestins (e.g., LNG) carried a lower risk of VTE than other EPs containing the third-generation progestins (e.g., desogestrel and gestodene) [38–40]. More recently, other important epidemiological data have shown LNG-containing EPs as being linked to a lower VTE risk than EPs containing other progestins such as gestodene, desogestrel, and drospirenone [41–45], also in comparison with nonoral route of administration [46]. For instance, in a 6-year cohort study, the following odds rations for VTE were reported: gestodene versus levonorgestrel, 1.86 (95% CI 1.59 to 2.18) desogestrel versus levonorgestrel, 1.82 (95% CI 1.49 to 2.22), and drospirenone versus levonorgestrel, 1.64 (95% CI 1.27 to 2.10) [42].

A possible explanation for this lower VTE risk with second-generation EPs (containing LNG) is that LNG exerts a stronger antiestrogenic effect at hepatic level than the other progestins for which a greater risk is reported, according to the concept that estrogenic component of EPs is the main reason for VTE risk in a dose-dependent manner. In addition, the progestin component may counteract this increased risk with different efficacy, according to progestin type (degree of residual androgenic and/or antiestrogenic effect of the progestin) [47]. Thus, third-generation and fourth-generation (drospirenone) progestins, not having androgenic or antiestrogenic action at hepatic level, may not sufficiently counteract the estrogen-dependent prothrombotic effect [48].

On the other hand, other studies report different results. In the EURAS study [49] and in the INGENIX study [50] the VTE risk with LNG-containing EPs did not differ from the VTE risk carried by drospirenone-containing EPs. In the Transatlantic Active Surveillance on Cardiovascular Safety of Nuvaring [51], Dinger et al. reported that etonogestrel-containing vaginal ring and other combined EPs (including EPs containing LNG) are associated with a similar risk of VTE; another study [52] showed no difference in VTE risk for norelgestromin-containing patch and etonogestrel-containing vaginal ring in comparison with low-dose estrogen comparators (including LNG-containing EPs).

Actually, VTE is one of the most serious side effects linked to the use of EPs, and even if rare, this condition can result in important consequences (in about 1-2% of all cases of VTE in women taking the pill) [53].

However, the absolute risk of having venous thromboembolism in EP users is low (the baseline risk is five per 100,000 person-years; this risk increases to about 15–25 per 100,000 person-years when taking the EP pill) [42, 55]. However, due to the very large number of EP users [56], even a small increase in this risk could affect a significant number of women. In any case, patients with a personal or family history of venous thromboembolism should not take combined EPs. Nevertheless, the European Medicines Agency (EMA), following the evaluation of epidemiological data, has yielded some documents in which it is declared that the LNG-containing EPs have a lower VTE risk in comparison with EPs containing other progestins with the same dose of EE (2001, regarding third-generation progestins, in particular gestodene and desogestrel) [57]; later, in 2005, another document of EMEA indicated LNG-containing EP pill as the reference standard to use as a comparator to evaluate the VTE risk for new contraceptive agents [58]. In 2011, a third document [59] reported also drospirenone-containing combined oral EPs as having a VTE risk higher than levonorgestrel-containing EPs (second generation EPs).

Recently, EMA [60] has indicated again LNG-containing EPs as being linked to a lower risk of VTE.

With respect to the risk of myocardial infarction and thrombotic stroke (arterial thromboembolism) linked to the use of EPs, it is well known that this risk is very rare among EPs users, and a recent work by Lidegaard et al. [61] reported no significant difference in myocardial infarction and thrombotic stroke risk according to progestin component among different EPs; rather, this risk appeared to be related to EE dosage, with the highest risk for EPs containing 50 mcg EE and the lowest risk for 20 mcg EE-containing EPs.

## 10. EE20/LNG100 and Acne

Thorneycroft et al. [54] evaluated the effects of EE20/LNG100 on androgen pattern and acne in 21 healthy women (age range: 18–28 years) in a 3-month study. EE20/LNG100 decreased significantly the levels of androgens (e.g., dehydroepiandrosterone sulphate, androstenedione, and total testosterone) in three compartments (adrenal, ovarian, and peripheral) and increased SHBG levels (Table 1). Total acne lesion count was reduced by the treatment with EE20/LNG100. Also in this population with signs of hyperandrogenism, the variation in body weight was not significant after 12 weeks of EE20/LNG100 administration, and blood pressure did not change: at baseline, mean systolic blood pressure (SBP) was 110 ± 11 mmHg and diastolic blood pressure (DBP) was 67 ± 9, while at the end of the study, SBP was 113 ± 13, and DBP 68 ± 8.

A randomized, multicenter, and placebo-controlled trial using EE20/LNG100 as an active treatment versus placebo [32] evaluated the efficacy of EE20/LNG100 in treating moderate acne over a 6-month period. Total, inflammatory and noninflammatory lesion counts at cycle 6 with EE20/LNG100 were significantly lower than with placebo; moreover, the EE20/LNG100 group had a better evaluation from clinicians and a better self-evaluation from patients versus the placebo group.

## 11. EE20/LNG100 and Sexuality

The EP use has been linked to a decreased sexual function [62]. It has been suggested that some women are more sensitive than others to a reduction of testosterone and free testosterone, showing a reduction in sexual interest [63, 64]. Moreover, a study by Coenen et al. [65] suggested that the decreased levels of free testosterone are due to the increased SHBG which, in turn, is determined by the estrogenic component of the EPs. EP, by blocking the ovulation, decreases further ovarian production of androgens. Thus, in premenopausal women, low level of androgens (in particular, free and total testosterone), associated with high level of SHBG, is believed to reduce sexual function during EP administration in women who previously had a normal sexual behavior [66, 67].

It is possible that an EP containing low levels of estrogen and/or a progestin retaining a partial residual androgenic (or antiestrogenic) activity counteracting the estrogen-induced SHBG increase less likely decreases sexual function in women [68]. On the other hand, it is not uncommon that a woman with an EP-induced decrease in sexual function is switched to an EP containing LNG as a progestin [69].

Interestingly, in a study [70] comparing two EPs containing EE and LNG differing between them only for the dosage of the two components (EE30/LNG150 versus EE 20/LNG100) about the effects on plasma androgen levels and sexual function over six cycles of administration, sexual function was evaluated at baseline and at the end of the study with Female Sexual Function Index (FSFI); moreover, also total testosterone (*T*) and SHBG were measured at baseline and at the end of the study. Free androgen index (FAI) was calculated as *T* (nmol/L) × 100/SHBG (nmol/L). *T* and FAI decreased in both groups, while SHBG increased. *T* and FAI were higher in EE20/LNG100 group in comparison with EE30/LNG150, and SHBG was lower. In particular, in EE30/LNG150 group, testosterone and FAI decreased by 32% and 67%, respectively, while SHBG increased by 32% (*P* < 0.05). On the other hand, in EE20/LNG100 group, *T* decreased by 20%, FAI decreased by 42%, and SHBG increased by 22%. Total score of FSFI did not differ between the two groups, but, over time, only in the EE20/LNG100 group a significant improvement was reported. These results could be explained by the low dose of EE and residual small androgenic activity of LNG and may be important in the overall clinical judgment on woman's health when EE20/LNG100 is administered.

## 12. Conclusions

EE20/LNG100 is a combination generally safe and well tolerated [31, 35]. The AEs reported for EE20/LNG100 are similar to those reported for other low-dose EPs [71]. Interestingly, in a study by Coney et al. [31] the percentage of women reporting one or more AEs is not different between EE20/LNG100 and placebo group.

Moreover, cycle control is effective [22, 26, 30, 33, 34], and body weight and body composition do not display any significant variation in various studies [20, 25]. This combination shows a mild or no significant effect from a metabolic (i.e., lipid and glucose metabolism) point of view [22–24].

Lastly, EE20/LNG100 has a low VTE risk and is considered as a gold standard by the European Regulatory Authorities in evaluating new EPs for this risk [58].

Overall, this favorable clinical profile of EE20/LNG100 can be considered in terms of safety, tolerability, and compliance in the process of individualization of EPs choice.

## Figures and Tables

**Table 1:** Percent (baseline versus end of the study) changes in androgens and SHBG levels over a 3-month period of treatment with EE20/LNG100 (modified from [54]).

Parameter	% change versus baseline	*P* (baseline versus end of treatment)
DHEAS (mcg/mL)	−18.9 ± 40.2	<0.05
Androstenedione (ng/mL)	−36.9 ± 26.7	<0.05
Total testosterone (ng/dL)	−27.0 ± 21.5	<0.05
3-androstanediol glucuronide (ng/mL)	−38.8 ± 36.1	<0.05
SHBG (nmol/L)	106 ± 89	<0.05
